# Supplementary material for: Learning Objectives Matrix in DIM.RUHR: A Didactic Concept for the Interprofessional Teaching of Data Literacy in Outpatient Health Care
Source: Healthcare (Basel). 2025 Mar 18;13(6):662. doi: 10.3390/healthcare13060662 (PMC11942240; doi:10.3390/healthcare13060662)
Supplement: Supplementary file 1 [file healthcare-13-00662-s001.zip › healthcare-3461999-supplementary.pdf]

Table S1: Learning Objectives Matrix for the Promotion of Data Skills in the Interprofessional Use of Health Data

Overview of the integrated subject areas

**Fundamentals and general concepts**	Basis understanding of data
	General principles and concepts of research data management (RDM)
	Research data policies
	Data management plans (DMPs)
	FAIR-Principles
	Open X (Open Data, Open Source, Open Science, …)
**Ethical, legal, and social considerations**	General legal aspects
	Data protection and personal data
	Special considerations for health/patient data
	Critical thinking
**Establishing a data culture**	Identifying data applications
	Specifying data applications
	Coordinating data applications
**Acquire data**	Modeling data applications
	Data collection and curation
	Data evaluation and ensuring data quality
**Managing data**	Data structure
	Data manipulation
	Data preparation
	Meta data and meta data standards
	Data protection and data maintenance
	Data storage and restoriation
	Publication paths for data; repositories
**Analyze data**	Data tools
	Evaluate data
**Interpret data**	Data comprehension
	Present data
**Derive actions**	Identify opportunities for action
	Evaluate impact

Abbreviations

Acronym	Full form
RDM	Research Data Management
DMP	Data Management Plan
FAIR (principles)	Findable, Accessable, Interoperable, Reusable
CARE (principles)	Collective Benefit, Authority to Control, Responsibility, Ethics
PID	Persistent Identifier
RAID	Redundant Array of Independent Disks

Subject area „Fundamentals and general concepts“

Learners can…

Fundamentals and general concepts	Learning objectives competence level 1 (Basic)	Learning objectives competence level 2 (Intermediate)	Learning objectives competence level 3 (Advanced)	Learning objectives competence level 4 (Highly specialized)
**Basic understandig of data**	… identify simple data types [K].<br>… understand and explain the difference between data, information, and knowledge [K].<br>… describe the difference between quantitative and qualitative data [K].	… explain what a data standard is [K].<br>… create personal data in reusable formats under guidance or convert them into appropriate formats [S].<br>… under guidance, assign appropriate procedures for quantitative and qualitative data [K, A, S].	… assess the characteristics of different data types, particularly regarding their processing [K].<br>… independently create personal data in reusable formats or convert them into appropriate formats [S].<br>… independently select appropriate procedures for quantitative and qualitative data [K, S].	… prepare the differences between data, information, and knowledge according to needs [K, S].<br>… design methods for automatic data import into various formats [K, S].<br>… evaluate the benefits/functions of data standards [K, A].<br>… derive difficulties in establishing data standards [K, A].<br>… provide guidance on basic inquiries regarding existing data standards and appropriate procedures for various data, or refer to designated personnel as needed [K, S].
**General principles and concepts of research data management (RDM)**	... describe the relevance and advantages/disadvantages of RDM for research [K, A].<br>... define research data and identify various forms of research data [K].<br>... describe the RDM cycle and identify its stages [K].	... identify measures for good RDM practices at various stages of the RDM cycle [K, A].<br>... explain the relevance and advantages/disadvantages of RDM [K, A].<br>... identify common issues in RDM [K, A].<br>... relate personal activities in a research process to the stages of the RDM cycle [K].	... independently select and justify measures for good RDM practices at various stages of the RDM cycle [K, A].<br>... critically question general and specific issues in RDM [K, A].<br>... communicate what RDM entails and why it is important for research processes [K, A, S].<br>... communicate which measures for good RDM practices are important at the stages of the RDM cycle [K, A, S].	... provide guidance on appropriate measures for good RDM practices at the stages of the RDM cycle [K, A, S].<br>... provide orientation on various aspects of RDM [K, S].<br>... advise on the relevance of RDM for good scientific practice [K, A, S].<br>... derive issues in RDM and develop solution strategies [K, A, S].

**Research data policies**	... describe what a research data policy is [K].<br>... describe the function of a research data policy [K].	... explain the difference between disciplinary and institutional research data policies [K].<br>... distinguish various forms of research data policies [K].<br>... apply research data policies to their own research projects under guidance [K, A, S].	... compare different research data policies and communicate their key contents to others [K, A, S].<br>... compare subject-specific policies and communicate their key contents to others [K, A, S].<br>... critically discuss research data policies [K, A].<br>... compare important policies and critically discuss their contents [K, A].<br>... independently implement and apply research data policies in their own research projects [K, A, S].	... evaluate information on guidelines and policies and prepare it according to the needs of various target groups [K, A, S].<br>... evaluate relevant policies and derive an action framework for RDM [K, A, S].<br>... develop new research data policies [K, S].
**Data management plans (DMPs)**	... describe what a DMP is [K].<br>... list the components of a DMP [K].<br>... describe the relevance of a DMP as a planning tool [K, A].	... create simple DMPs for their own projects under guidance [K, S].	... assess and critically discuss the relevance and concept of a DMP [K, A].<br>... independently create a DMP according to funding guidelines [K, S].<br>... use tools to create DMPs for their own projects [K, S].<br>... critically discuss the advantages and disadvantages of a DMP [K, A].	... determine the criteria by which funding applications are evaluated [K, A].<br>... provide information about tools for creating DMPs and advise on their use [K, S].<br>... advise on the creation of (project-specific) DMPs [K, S].<br>... incorporate best practice examples of DMP use from researchers' perspectives [K].<br>... evaluate national and international perspectives on DMPs [K, A].<br>... implement different forms of project management and combine them with the DMP in their own research [K, S].
**FAIR-Principles**	... name the FAIR principles [K].<br>... describe the individual aspects of FAIR [K].	... identify the advantages and disadvantages of FAIR data [K, A].	... communicate the FAIR principles to others [K, S].<br>... weigh and communicate the pros, cons, and challenges of	... evaluate the FAIRness of data [K, A].<br>... provide guidance and project-specific advice on data

		... assess the FAIRness of data [K, A].<br>... integrate the FAIR principles into their own research projects in an application-oriented manner under guidance [K, A, S].	data FAIRification to others [K, A, S].<br>... independently integrate the FAIR principles into their own research projects in an application-oriented manner [K, A, S].	FAIRification [K, S].
**Open X (Open Data, Open Source, Open Science, …)**	... define the concept of "Open Science" [K].<br>... define the terms Open Access, Open Data, and Open Source as areas or practices within Open Science in terms of their reproducibility [K].<br>... name the advantages and disadvantages of Open Science and related practices [K, A].	... differentiate between the concepts of "FAIR" and "Open" [K].<br>... under guidance, assess whether a publication is freely accessible [K].<br>... under guidance, find platforms for open data [S].<br>... assess what is required to make research data openly accessible [K].<br>... apply Open Science tools in their own research process or professional practice under guidance [S].<br>... publish their own data in the context of Open Science under guidance [S].	... assess what opening the research cycle means in the context of Open Science [K, A].<br>... weigh the challenges of opening the research cycle in the context of Open Science [K, A].<br>... compare which Open Science practices are applicable for individual steps in an exemplary research cycle [K, A].<br>... assess the degree of openness of data using the 5-star Open Data model of the European Commission [K, A].<br>... independently apply Open Science tools in their own research process or professional practice [S].<br>... independently publish their own data in the context of Open Science [S].	... evaluate what opening the research cycle means specifically (in individual stages of the research cycle) in the context of Open Science [K, A].<br>... provide guidance and project-specific advice on Open Science practices [K, S].

Coding of competency areas: Knowledge [K], skills [S] and attitudes [A]

Subject area „Ethical, legal, and social considerations“

Learners can…

Ethical, legal and social considerations	Learning objectives competence level 1 (Basic)	Learning objectives competence level 2 (Intermediate)	Learning objectives competence level 3 (Advanced)	Learning objectives competence level 4 (Highly specialized)
**General legal aspects**	... demonstrate the role of copyright and licensing in research data management (FDM) [K].<br>... name relevant licensing systems [K].<br>... describe the characteristics of open and restrictive licenses [K].	... under guidance and adhering to legal and ethical guidelines, use available software and hardware (PC, programs, internet, etc.) appropriately [K, A, S].<br>... under guidance, select an appropriate license for publishing their own research data [K, A, S].	... independently and adhering to legal and ethical guidelines, use available software and hardware (PC, programs, internet, etc.) appropriately [K, A, S].<br>... apply copyright rules to their research project [K, S].<br>... independently select an appropriate license for publishing their own research data [K, A, S].<br>... compare and communicate the different levels of legal regulations significant to FDM [K, A, S].	... determine and provide guidance on the legal aspects to consider at different stages of the research cycle, advising and training accordingly [K, A, S].<br>... determine the legal aspects necessary for an Open Science publication [K, A].<br>... provide guidance regarding legal aspects of an Open Science publication [K, S].<br>... provide basic guidance on copyright and licensing inquiries and refer to designated personnel if needed [K, S].<br>... evaluate current legal developments related to FDM and provide foundational training [K, A, S].
**Data protection and personal data**	... define what personal data and sensitive data are and provide examples [K].<br>... name reasons for the special protection of personal data [K, A].<br>... name the general legal foundations for the protection of personal data [K].<br>... demonstrate general data protection measures (e.g., passwords, etc.) [K].<br>... name methods and measures to protect the identity of research participants [K].	... assess whether research data contain personal data [K, A].<br>... assess which personal data may be collected [K, A].<br>... under guidance, apply suitable methods to protect the identity of research participants [K, S].<br>... under the guidance of a data protection officer, create informed consent for their own research [S].<br>... under guidance, apply methods for legally secure anonymization and pseudonymization to their own data [S].	... independently select and apply suitable methods to protect the identity of research participants for their own research [K, A, S].<br>... independently assess whether informed consent is required for their research project [K, A].<br>... explain the content and significance of informed consent to others [K, S].<br>... independently draft informed consent for their own research projects after consultation with a data protection officer [K, S].	... provide guidance on basic data protection inquiries and refer to designated personnel if needed [K, S].<br>... provide guidance on the content of informed consent [K, S].<br>... design templates for informed consent [K, S].<br>... provide guidance on assessing the degree of anonymizability of research data based on information content [K, S].

	... demonstrate who is liable in case of a breach of data protection regulations [K].<br>... outline the consequences of a breach of data protection regulations [K].<br>... outline the notification requirements applicable to the collection of personal data for research purposes [K].<br>... describe the content and significance of informed consent [K, A].<br>... outline the definitions and differences between "anonymization" and "pseudonymization" [K].<br>... present various methods for legally secure anonymization and pseudonymization of data [K].	... under the supervision of a data protection officer, identify existing weaknesses and risks regarding data protection in professional practice [K, A, S].<br>... under the guidance of a specialized company, establish new data protection structures within their own organization [S].	... independently apply various methods for legally secure anonymization and pseudonymization of data to their own data [K, S].<br>... compare various methods for legally secure anonymization and pseudonymization of data and communicate their fundamentals to others [K, A, S].<br>... independently assess the degree of anonymizability of their data based on information content [K, A].<br>... independently establish new data protection structures within their own organization [S].	
**Special considerations for health/patient data**	... describe the legal foundations of medical confidentiality [K].<br>... describe the requirements for medical documentation [K].<br>... outline the additional requirements in data protection for health data [K].<br>... outline the significance of an ethics vote [K, A].	... demonstrate the legal foundations of medical confidentiality in their own (research) situation under guidance [K, S].<br>... create medical documentation under guidance [K, S].<br>... apply the additional requirements in data protection for health data under guidance in their own (research) situation [K, S].<br>... appropriately document patient data under guidance [K, S].	... convey the legal foundations of medical confidentiality [K, S].<br>... independently draft a medical documentation [K, S].<br>... independently apply the additional requirements in data protection for health data in their own (research) situation [K, A, S].<br>... independently document patient data appropriately [K, S].	... provide guidance on the legal foundations of medical confidentiality [K, S].<br>... provide guidance to others regarding the additional requirements in data protection for health data and refer to designated personnel if needed [K, S].

		... explain data to patients while considering and adhering to ethical guidelines [K, A].		
**Critical thinking**	... outline (higher-level) problems and challenges related to data [K].<br>... name problems and list possible solutions for emerging issues [K].	... identify and assess (higher-level) problems and challenges related to data under guidance [K].<br>... apply solution strategies under guidance [K, S].	... assess (higher-level) problems and challenges related to data [K, A, S].<br>... independently select and apply solution strategies [K, A, S].	... devise an appropriate solution plan for project-specific problems and challenges [K, A, S].<br>... provide guidance on higher-level problems and challenges related to data and offer project-specific advice or refer to designated personnel if needed [K, A, S].

Coding of competency areas: Knowledge [K], skills [S] and attitudes [A]

Subject area „Establishing a data culture“

Learners can…

Establishing a data culture	Learning objectives competence level 1 (Basic)	Learning objectives competence level 2 (Intermediate)	Learning objectives competence level 3 (Advanced)	Learning objectives competence level 4 (Highly specialized)
**Identifying data applications**	... define the term "data culture" [K].<br>... outline potential value contributions of data and their limitations [K, A].<br>... illustrate the possibilities of using data for learning, research, and decision-making [K].<br>... understand the potentials of data literacy and data culture at individual and systemic levels [K, A].	... identify knowledge gaps and background information (conditions, context, etc.) relevant to one's (research) topic [K].<br>... distinguish between irrelevant and relevant information within the data-related system [K].	... communicate awareness of the benefits of sustainable and open data culture as well as existing structures to others [K, A, S].<br>... transparently formulate a specific, data-driven task based on identified knowledge gaps and background information [K, S].	... provide guidance on potential data applications and offer project-specific advice [K, S].<br>... evaluate existing rules and process flows [A].
**Specifying data applications**	... describe the measurability of objects with examples [K].<br>... outline minimal requirements for data-related activities [K].	... structure a process flow into objects and their relationships under guidance [S].	... formulate optional requirements for data-related activities [K, S].<br>... independently derive hypotheses about the	... provide project-specific advice and guidance or refer to interdisciplinary advisory services regarding specific requirements [K, S].

	... outline possible use cases for data utilization with examples [K].	... formulate hypotheses about the relationships between data under guidance [S].	relationships between data [K, S].<br>... transparently communicate the strategy for establishing a data culture to others [K, S].	
**Coordinating data applications**	... name basic techniques of project management [K].	... conduct the planning and coordination of a data project under guidance [S].<br>... use collaboration and versioning tools under guidance [S].	... independently and transparently carry out the planning and coordination of a data project [K, S].<br>... assess and integrate the different requirements of other project stakeholders into the data project [K, A, S].<br>... interdisciplinarily communicate information to stakeholders [K, S].<br>... mediate interdisciplinarily between stakeholders [K, S].<br>... independently use collaboration and versioning tools [S].	... design the planning and coordination of a data project in specialized application settings [S].<br>... advise others on the use and adoption of collaboration and versioning tools [K, S].<br>... keep oneself and others informed about the latest techniques and tools of project and data management [S].

Coding of competency areas: Knowledge [K], skills [S] and attitudes [A]

Subject area „Acquire data“

Learners can…

Acquire data	Learning objectives competence level 1 (Basic)	Learning objectives competence level 2 (Intermediate)	Learning objectives competence level 3 (Advanced)	Learning objectives competence level 4 (Highly specialized)
**Modeling data applications**	... define what a variable is [K].<br>... indicate possible biases and information losses [K].	... convert process models into data models under guidance [S].<br>... assess under guidance which questions require what type of data [K, S].	... independently convert process models into data models [S].<br>... independently assess which questions require what type of data [K, S].	... incorporate different perspectives in the development of the data model [S].<br>... guide selection and evaluation processes to determine which information is

				needed for the question [K, A, S].
**Data collection and curation**	... list various data collection methods [K].<br>... name characteristics of data based on the collection method [K].<br>... define primary and secondary data [K].<br>... demonstrate the function and importance of databases [K, A].<br>... illustrate the importance of evidence-based research [K, A].	... conduct various data collection methods under guidance [S].<br>... identify suitable data sources for the research question [K].<br>... conduct literature data research under guidance [S].<br>... merge data from different databases under guidance [S].<br>... present best practices in health services research under guidance [K].<br>... structure data into tables under guidance for efficient utilization by analytical tools [S].	... independently conduct various data collection methods [S].<br>... educate others on various data collection methods [K, S].<br>... independently conduct literature data research [S].<br>... independently merge data from different databases [S].<br>... perform data integration into various formats [S].	... design a suitable data collection strategy for specific research questions [K, S].<br>... evaluate novel data sources such as Big Data and wearables [K, A].
**Data evaluation and ensuring data quality**	... define data quality and its parameters [K].<br>... list various aspects of data quality (content-related, context-related, presentation-related, accessibility-related) [K].<br>... name measures for improving data quality [K].<br>... describe strategies for error prevention in handling data [K].	... identify qualitative deficiencies (accuracy, relevance, representativeness, completeness) in own and others' data [K, A].<br>... document the systematic review of data under guidance [S].<br>... explain strategies for error prevention in handling data and apply them under guidance [K].	... independently correct identified quality deficiencies in their own data [S].<br>... assess data quality based on its intended use [A].<br>... create data according to standards of good data quality [S].<br>... convey measures for improving data quality to others [K, S].	... determine data quality [K, A].<br>... develop strategies and processes for improving data quality and provide guidance on this [K, A, S].

Coding of competency areas: Knowledge [K], skills [S] and attitudes [A]

Subject area „Managing data“

Learners can…

Managing data	Learning objectives competence level 1 (Basic)	Learning objectives competence level 2 (Intermediate)	Learning objectives competence level 3 (Advanced)	Learning objectives competence level 4 (Highly specialized)

**Data structuring**	... describe the relevance of structured data naming [K, A].<br>... name criteria for a good naming convention [K].<br>... describe the purpose and benefits of versioning [K, A].<br>... name methods and tools for version management [K].<br>... recognize why raw data should be stored separately [K].<br>... show what functions data documentation fulfills [K, A].<br>... describe the components/contents of data documentation [K].<br>... describe what a PID (Persistent Identifier) is [K].<br>... outline the role of ontologies and controlled vocabularies [K].<br>... explain what a relational database is [K].	... under guidance, create naming conventions and apply them to own (and collaboratively used) research data [S].<br>... under guidance, apply simple versioning methods to own research data [S].<br>... using examples, explain the advantages and disadvantages of different documentation forms [K].<br>... under guidance, formulate simple data documentation for own research data [S].<br>... under guidance, use PIDs for retrieval and referencing purposes [S].<br>... place the use of ontologies and controlled vocabularies in context under guidance [K].<br>... categorize domain-relevant ontologies as such under guidance [K].<br>... structure data under guidance [S].<br>... explain the connection between relational databases [K].	... independently create naming conventions and apply them to own (and collaboratively used) research data [S].<br>... apply versioning methods and tools for collaborative work and automation of shared workflows independently [S].<br>... independently create simple data documentation [S].<br>... create PIDs for personal data and collaborative work independently [S].<br>... apply ontologies and controlled vocabularies to describe resources independently [S].<br>... independently structure data [S].	... evaluate various concepts for creating naming conventions and provide consulting support to third parties regarding the development of naming conventions [K, A, S].<br>... inform and advise third parties on simple and complex versioning methods and tools [K, A, S].<br>... independently design complex data documentation for own research data [F].<br>... design a traceable workflow for data documentation and advise others on this [K, A, S].<br>... develop templates for data documentation [K, S].<br>... develop ontologies and controlled vocabularies [S].<br>... provide project-specific consulting support regarding the development of ontologies and controlled vocabularies [K, S].
**Data manipulation**	... list various file formats [K].<br>... outline processes of standardization, compression, or conversion [K].<br>... name levels of measurement (scale) for data [K].	... clean data technologically under guidance [S].<br>... differentiate between various types of data compression (lossless, lossy) [K].<br>... convert data under guidance through standardization, compression, or conversion [S].<br>... merge different data sources technologically under guidance [S].	... independently correct data [F].<br>... independently convert data through standardization, compression, or conversion [F].	... provide guidance and consulting support on structural/syntactic data cleaning [K, F].

		... assess the alteration of information content in data due to transformation processes [K, A].		
**Data preparation**	... describe the relevance of (complex) data preparation [K, A].<br>... define linking processes [K].<br>... recognize outliers and anomalies [K, S].	... transform data under guidance through aggregation or combination [S].	... independently transform data through aggregation or combination [S].<br>... weigh the alteration of information content in data due to transformation processes [K, A].	... provide guidance and consulting support on substantive/semantic data cleaning [K, S].
**Metadata and meta data standards**	... define the terms "metadata," "PID," and "controlled vocabulary" [K].<br>... describe the benefits of using metadata [K].<br>... define what a metadata standard is [K].<br>... list the functions of metadata (especially for data reuse) [K].	... list various metadata standards [K].<br>... find metadata standards under guidance [S].<br>... explain data based on given, simple standards with metadata [K].<br>... use metadata under guidance [S].	... independently research metadata standards [S].<br>... independently assess data based on existing standards [A].<br>... explain the different metadata standards that exist [K, S].<br>... explain the use cases for metadata standards [K, S].	... appropriately prepare common controlled vocabularies for metadata [K, S].<br>... determine suitable (disciplinary-specific) metadata standards [K, A].<br>... include relevant initiatives and projects on the topic [K, S].<br>... provide project-specific advisory support regarding the selection of metadata standards [K, A, S].
**Data protections and data maintenance**	... name general data maintenance requirements (e.g., storing, deleting, updating, etc.) [K].<br>... name general data protection requirements (e.g., retention schedules, accessibility, requirements for sharing, protected drives, etc.) [K].	... explain the use of policies regarding data protection and access control [K].<br>... reflect data maintenance requirements on their own data [K].<br>... apply data protection requirements under guidance to their own data [K].	... apply data maintenance requirements to their own data [S].<br>... independently apply data protection requirements to their own data [S].	... respond to inquiries regarding data maintenance and provide guidance to others [K, S].<br>... respond to inquiries regarding data protection and provide guidance to others [K, S].
**Data storage and restoriation**	... understand the retention policies for health data [K].<br>... list various storage systems and media [K].<br>... describe what a backup is [K].	... assess which storage systems and media are suitable for specific requirements and scenarios [K, A].	... select appropriate storage systems and media for given requirements and scenarios [K, A].<br>... create and apply their own backup strategy [S].	... determine the most suitable storage systems and media for specific requirements and scenarios [K, A, S].<br>... evaluate the advantages and risks of different storage

	... name why backups should be used [K, A].<br>... describe the 3-2-1 backup rule for data backup in your own words [K].<br>... list criteria for selecting research data for archiving [K, A].<br>... describe special requirements for archiving research data [K].<br>... describe what a RAID system is [K].<br>... show why RAID systems should be used [K, A].	... create and apply their own backup strategy under guidance [S].<br>... differentiate between various institutional backup solutions [K].<br>... apply the 3-2-1 backup rule to their own data under guidance [S].<br>... differentiate between various methods for long-term data archiving [K].<br>... outline institutional solutions (repositories) [K].<br>... apply institutional solutions under guidance [S].<br>... explain the differences between various RAID systems [K].	... assess the necessity of using a RAID system [K, A].	systems, media, and storage organizations in specific contexts [K, A].<br>... derive questions regarding the sustainability of research data [K, A].<br>... provide project-specific advice on suitable repositories for archiving research data [K, A, S].<br>... assist other researchers in utilizing existing storage systems, media, and backup strategies [K, S].
**Publication paths for data; repositories**	... describe what data publication is [K].<br>... list components of a data publication [K].<br>... list various publication paths for data [K].<br>... define what a repository is [K].<br>... list different types of repositories [K].<br>... list advantages and disadvantages of different data repositories [K, A].	... explain the advantages and disadvantages of data publication [K].<br>... present own data under guidance [S].	... independently select suitable repositories for long-term archiving [S].<br>... assess and evaluate the appropriateness of data publication in specific cases [K, A].<br>... independently publish data [S].<br>... formulate criteria for selecting a repository [A, S].<br>... independently select repositories for data reuse and publication of own data [S].	... provide project-specific advice on the advantages and disadvantages of different data publication avenues [K, A, S].<br>... guide others in independently publishing data [S].<br>... provide guidance on important technical and organizational requirements for operating repositories and refer or direct to specialized personnel [K, A, S].

Coding of competency areas: Knowledge [K], skills [S] and attitudes [A]

Subject area „Analyze data“

Learners can…

Analyze data	Learning objectives competence level 1 (Basic)	Learning objectives competence level 2 (Intermediate)	Learning objectives competence level 3 (Advanced)	Learning objectives competence level 4 (Highly specialized)
**Data tools**	... name different helpful tools for data management (in different research phases) [K].<br>... name suitable software for the evaluation of data [K].	... use different helpful tools for data management under guidance (in different research phases) [S].<br>... research tools according to their individual requirements and tasks under guidance [S].	... weigh up which tools are particularly useful [K, A].<br>... independently use various helpful tools for data management (in different research phases) [S].<br>... independently analyze (self-collected) data with the help of suitable software [S].	... provide advice and training on various helpful tools for data management (in different phases of research) [K, S].<br>... provide project-specific and individual requirements and tasks with regard to suitable tools [K, A, S].
**Evaluate data**	... describe descriptive and inferential statistics [K].<br>... define significance [K].<br>... name requirements for parametric test procedures [K].<br>... name prerequisites for parametric test procedures [K].	... apply descriptive-statistical methods under guidance [S].<br>... apply inferential statistical methods under guidance [S].<br>... carry out qualitative data analysis under supervision [S].<br>... apply suitable analysis methods based on the respective task [S].	... apply descriptive-statistical methods independently [S].<br>... apply inferential statistical methods independently [S].<br>... carry out qualitative data analysis independently [S].	... in a programming language develop their own evaluation programs [K, S].<br>... design their own analysis plans [K, S].

Coding of competency areas: Knowledge [K], skills [S] and attitudes [A]

Subject area „Interpret data“

Learners can…

Interpret data	Learning objectives competence level 1 (Basic)	Learning objectives competence level 2 (Intermediate)	Learning objectives competence level 3 (Advanced)	Learning objectives competence level 4 (Highly specialized)
**Data comprehension**	… describe statistical parameters [K].<br>... show the relationship between different key figures [K].<br>... name the difference between correlation and causality [K].	... explain data products (statistics and model results) and graphics [K].<br>... research relevant data for their own projects under supervision [S].<br>... reproduce reused data correctly [W].	... interpret data products (statistics and model results) and graphics independently [S].<br>... compare different sources of information, research tools and strategies for researching data and communicate them to third	... implement and evaluate different sources of information, research tools and strategies for researching data in their own research [K, A].<br>... combine different information from various sources and derive

	... name sources of information for researching research data [K].	... assess the trustworthiness of a data source [W, H].<br>... research data under supervision [S].<br>... apply rules for citing research data under guidance [S].	parties in a target group-oriented manner [K, S].<br>... formulate rules for citing data and communicate them to third parties in a way that is appropriate for the target group [K, S].	new findings [K, A, S].
**Present data**	... define various specialist statistical terms [K].<br>... describe different presentation methods in a comprehensible way [K].	... present results under guidance using statistical terminology [S].<br>... select under guidance which information is relevant for the presentation of results [S].<br>... present results visually under guidance [S].<br>... produce a scientific publication under guidance [S].	... communicate results clearly and coherently to non-specialists [K, S].<br>... present the results in a comprehensible and understandable way for everyone, regardless of the time available [K, S].<br>... independently present results visually [S].<br>... independently produce a scientific publication [S].	... educate others about the information according to the background knowledge of the addressee [K, A, S].

Coding of competency areas: Knowledge [K], skills [S] and attitudes [A]

Subject area „Derive actions“

Learners can…

Derive actions	Learning objectives competence level 1 (Basic)	Learning objectives competence level 2 (Intermediate)	Learning objectives competence level 3 (Advanced)	Learning objectives competence level 4 (Highly specialized)
**Identify opportunities for action**	... outline important cornerstones of domain knowledge [K].	... distinguish the information obtained from the data [K].<br>... derive possible measures from the data under guidance [K, A, S].	.. question existing rules and processes [A].<br>... communicate topic-relevant, interdisciplinary knowledge to others [K, S].<br>... weigh up different measures against each other [K, A].	... design complete action plans based on the information obtained and possible value contributions [K, S].<br>... determine decisions/solutions and/or provide orientation [K, A, S].

**Evaluate impact**	... describe the relevance of follow-up surveys for assessing the effectiveness of decisions/solutions [K, A].<br>... list possible evaluation approaches [K].	... create an evaluation plan under guidance [S].	... independently create an evaluation plan [S].<br>... independently collect and evaluate follow-up data [S].<br>... analyze collected follow-up data and compare it with original results or other studies [S, A].	... evaluate the decisions/ solutions based on the data and implement new decisions/ solutions if necessary [K, A, S].<br>... guide and/or provide orientation for a continuous improvement process [K, A, S].

Coding of competency areas: Knowledge [K], skills [S] and attitudes [A]
